# Clinical Trial Validation of Automated Segmentation and Scoring of Pulmonary Cysts in Thoracic CT Scans

**DOI:** 10.3390/diagnostics14141529

**Published:** 2024-07-15

**Authors:** Aneesha Baral, Simone Lee, Farah Hussaini, Brianna Matthew, Alfredo Lebron, Muyang Wang, Li-Yueh Hsu, Joel Moss, Han Wen

**Affiliations:** 1 Laboratory of Imaging Physics, Biochemistry and Biophysics Center, National Heart, Lung and Blood Institute, National Institutes of Health, Bethesda, MD 20892, USA; aneesha.baral@nih.gov (A.B.); simone.lee@nih.gov (S.L.); farah.hussaini@nih.gov (F.H.); brianna.matthew@nih.gov (B.M.); alfredolebron08@gmail.com (A.L.); muyang.wang2@nih.gov (M.W.); 2 Department of Radiology and Imaging Sciences, Clinical Center, National Institutes of Health, Bethesda, MD 20892, USA; lyhsu@nih.gov; 3 Pulmonary Branch, National Heart, Lung and Blood Institute, National Institutes of Health, Bethesda, MD 20892, USA; mossj@nhlbi.nih.gov

**Keywords:** lymphangioleimyomatosis, cystic lung disease, CT score, cyst burden, cyst score, low-attenuation volume, emphysema

## Abstract

In cystic lung diseases such as lymphangioleiomyomatosis (LAM), a CT-based cyst score that measures the percentage of the lung volume occupied by cysts is a common index of the cyst burden in the lungs. Although the current semi-automatic measurement of the cyst score is well established, it is susceptible to human operator variabilities. We recently developed a fully automatic method incorporating adaptive features in place of manual adjustments. In this clinical study, the automatic method is validated against the standard method in several aspects. These include the agreement between the cyst scores of the two methods, the agreement of each method with independent tests of pulmonary function, and the temporal consistency of the measurements in the consecutive visits of the same patients. We found that the automatic method agreed with the standard method as well as the agreement between two trained operators running the same standard method; both methods obtained the same level of correlation with laboratory pulmonary function tests; the automated method had better temporal consistency than the standard method (*p* < 0.0001). The study indicates that the automatic method could replace the standard method and provide better consistency in assessing the extent of cystic changes in the lungs of patients.

## 1. Introduction

Lymphangioleiomyomatosis (LAM) is a rare, progressive lung disease that affects 3.4–7.8 women per million [1]. It is caused by mutations in the tumor-suppressing tuberous sclerosis complex (TSC), leading to the constant activation of the mechanistic target of rapamycin (mTOR) biochemical pathway [2,3]. This results in the proliferation of smooth muscle cells that can lead to the formation of air-filled cysts in the lungs due to airway obstruction, airway narrowing, and air trapping [3]. There are two types of LAM: TSC-LAM and sporadic LAM. TSC-LAM is a hereditary form of the disease that results from germline mutations in TSC genes [2]. Sporadic LAM, the less common form, occurs due to somatic mutations primarily in TSC 2 and predominantly affects premenopausal women [4]. The clinical features of LAM disease include recurrent pneumothorax, chylous effusions, and shortness of breath caused by airflow obstruction and hyperinflation [5]. Most patients with LAM have declines in pulmonary function tests (PFTs) including a decline in airflow due to an increase in airway resistance and poor gas exchange via a reduction in diffusion capacity [3]. Although LAM primarily affects the lungs, it can also have multiorgan manifestations such as epilepsy, brain lesions, and renal angiomyolipoma [6].

**Figure 1:**
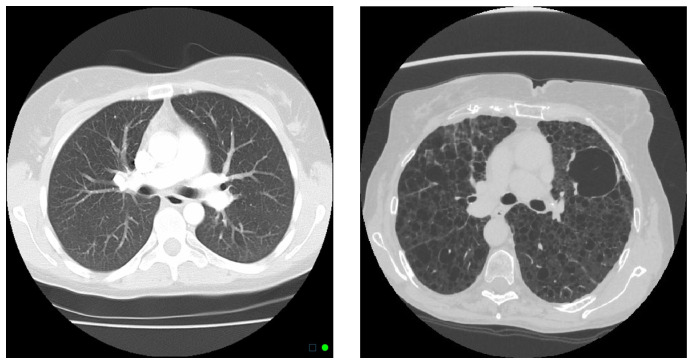
Comparing the thoracic CT scan of healthy lungs (**left**) and lungs with LAM (**right**). In the image on the right, round, air-filled cysts of a range of sizes appear as dark voids in the lung tissue.

Given that patients in the US have a median transplant-free survival of 29 years, regular monitoring is an important part of the treatment and management of the disease [7,8]. Traditionally, pulmonary function tests have been used to monitor lung function with an emphasis on metrics such as forced expiratory volume (FEV_1_), the Tiffeneau–Pinelli index of forced expiratory volume over forced vital capacity (FEV_1_/FVC), and diffusion capacity for carbon monoxide (DL_CO_) [9,10]. A more recent method to help in the diagnosis and prognosis of this rare disease has been the use of high-resolution computed tomography (CT). CT scans offer the direct visualization of cystic changes and are essential for generating the cyst score, a quantitative measure of the percentage of lung volume occupied by cysts (Figure 1). Studies have demonstrated the close association between cyst scores and declines in pulmonary function, offering clinicians an additional tool for managing the disease in patients with LAM [7,11,12,13]. 

Currently, the gold-standard methods to segment the cysts and calculate the cyst score are the FDA-approved semi-automatic software (e.g., Canon Medical Systems USA, Inc., Tustin, CA, USA). However, semi-automatic methods have inherent limitations. They rely on trained operators to make manual adjustments based on visual impressions to correct for instrumental and patient-to-patient variabilities. As a result, they are susceptible to inter- and intra-operator variability, leading to potential inconsistencies in the cyst score [14]. These affect the evaluation of the rate of the change or progression of the cyst burden in the lungs over time. Additionally, the requirement for operators to undergo training to maintain uniform visual standards limits the accessibility of the procedure. 

A solution to this problem is fully automatic cyst segmentation. Previously, Schmithorst and co-authors have described a method based on the frequency histogram of the CT values of the whole lung to automatically calculate the percentage volume occupied by cysts [15]. However, the method does not provide the identification and segmentation of the cysts in the images. More recently, we introduced a fully automatic method for generating cyst segmentation and cyst scores based on the CT attenuation values of the air space surrounding the body, the large airways, and the local parenchyma [14]. These calculations remove the need for the subjective visual adjustment of the attenuation threshold by the trained operator, thus removing the human influence in the process. The technical details of the method are described in a previous study [14]. 

In this clinical study, we assessed the automatic method in a cohort of patients with LAM by comparing it with the gold-standard semi-automatic method and correlating it with pulmonary function tests. Specifically, we evaluated the agreement between the measurements of the automatic and semi-automatic methods, the agreement of each method with the laboratory tests of pulmonary function, and the consistency of the measurements in consecutive visits by the same patients. 

## 2. Materials and Methods

### 2.1. Study Population 

We performed a retrospective study on 208 CT scans from 152 female patients with LAM at the Clinical Center of the National Institutes of Health (NIH), Bethesda, MD, USA during the period from June 2018 to May 2024. The patients were enrolled in the clinical research protocol “Role of Genetic Factors in the Pathogenesis of Lung Disease” (clinicaltrials.gov, NCT00001532), which was approved by the National Heart, Lung, and Blood Institute, the National Institutes of Health Institutional Review Board (IRB # 96-H-0100). The diagnosis of LAM was confirmed by the American Thoracic Society/Japanese Pulmonary Society criteria [10]. Ten patients had a history of tuberous sclerosis complex (TSC). The age range of the study population was from 25.9 to 76.0 years with a median age of 50.9 years. Some of the patients are followed at our hospital on a regular basis and have undergone CT scans at annual or bi-annual intervals as part of their regular visits.

### 2.2. Thoracic CT Scan Protocol 

All the patients received inspiratory volumetric high-resolution helical chest CT scans with a nominal breath-hold period of 10 s and a nominal dose of 0.16 rem. The reconstruction slice thickness was 1 to 2 mm and the slice center-to-center spacing was 1 mm. The in-plane pixel resolution was between 0.6 and 0.8 mm. Depending on the scanner platform, a soft-tissue type of convolution kernel was employed in the reconstruction.

### 2.3. Laboratory Pulmonary Function Tests

Pulmonary function tests (PFTs) were performed in a clinical pulmonary physiology laboratory of the hospital during the same visit as the CT exams. The PFT values that were utilized for this study were the forced expiratory volume in the first second, expressed in the percentage of predicted values (FEV_1__pp); FEV_1_ normalized to the forced vital capacity expressed in the percentage of predicted values (FEV_1_/FVC_pp); and the diffusion capacity of the lungs for carbon monoxide, adjusted for hemoglobin and expressed in percentage of predicted values (DL_CO__adj_pp) [16,17].

### 2.4. Standard Semi-Automatic Cyst Score Procedure

The FDA-approved software for cyst segmentation and cyst score calculation is part of the CT scanner platform (Canon Aquilion ONE, Canon Medical Systems USA, Tustin, CA, USA). Depending on the version of the scanner software and hardware, the chest CT series with the appropriate reconstruction setting is loaded into the software. The software makes an initial segmentation of the cystic areas based on a fixed global threshold of the CT attenuation value of −940 Hounsfield Units. A trained operator then inspects the segmentation and adjusts the threshold either upward or downward by the appropriate amount until a visually satisfactory segmentation is achieved. After segmentation, the software automatically sums up the total volume of the cysts and calculates the percentage ratio of total cyst volume/total lung volume as the cyst score. In this study, two trained operators used the FDA-approved software to generate standard scores for inter-operator comparison, as explained in Section 2.6.1 below.

### 2.5. Automatic Cyst Score Procedure

The automatic procedure was applied to the same CT image series as the standard semi-automatic procedure. The software uses established algorithms to automatically isolate the lung volumes and the large airways [18,19] before applying information from several sources, including the surrounding air background, the airways, and the local parenchyma to calculate the CT attenuation thresholds for cystic areas on a location-specific basis. The thresholds were then applied to automatically segment the cystic areas, and the software then calculated the percentage volume fraction of the lungs occupied by the cysts as the cyst score. A detailed description of the software pipeline is given by Lee and co-authors [14].

### 2.6. Statistical Analysis

#### 2.6.1. Comparing Automatic and Standard Cyst Scores

We assessed the inter-method difference between the standard semi-automatic procedure and the new automatic procedure, using as a reference the intra-method variability of the semi-automatic procedure itself. The differences between cyst scores generated by the semi-automated and automated methods were evaluated in 100 CT scans from 100 patients. These scans were accompanied by concurrent pulmonary function tests in the same visits. The differences between the two scores were analyzed in two ways: through the Bland–Altman analysis, which examines the mean and variance of the difference [20], and by assessing the mean and variance of the absolute difference. We then considered whether the differences between the automatic and standard scores could be accounted for by the human variability within the standard cyst score itself. For this purpose, we obtained the standard cyst scores from two trained operators scoring the same scans independently. These data were available for a separate set of 26 CT scans from a group of 19 patients. Consequently, two sets of differences were obtained from two pairs of cyst scores: one set between the automatic and standard cyst scores, the other between the standard cyst scores from the two operators. We compared the means and variances of the two differences with Welch’s t-test and the two-sample F-test, respectively. Since the first pair of scores covered a wider range of cyst scores than the second pair, it was truncated down to the same range for the comparison to be valid.

#### 2.6.2. Comparing the Correlation of the Cyst Scores to Pulmonary Function Tests

We obtained the correlations of the automatic and standard cyst scores with the three pulmonary function tests in the 100 CT scans described above. We then compared the two cyst scores in terms of their correlation with the PFTs using the test of the difference between two dependent correlations with one variable in common [21].

#### 2.6.3. Comparing the Consistency of the Cyst Scores from the Two Methods

The inconsistency of the cyst scores over time, whether due to operator inconsistencies, instrumental factors, or patient factors, will introduce stochastic fluctuations in the cyst scores resulting in a greater variance of the rate of the change in the cyst scores. With this reasoning, we studied the cyst score rate of change from the most recent two consecutive visits in a group of 41 patients. The median interval between the two visits was 15.0 months. We compared the automatic and the standard semi-automatic methods in terms of the average and the variance of the cyst score rate of change.

## 3. Results

**Figure 2:**
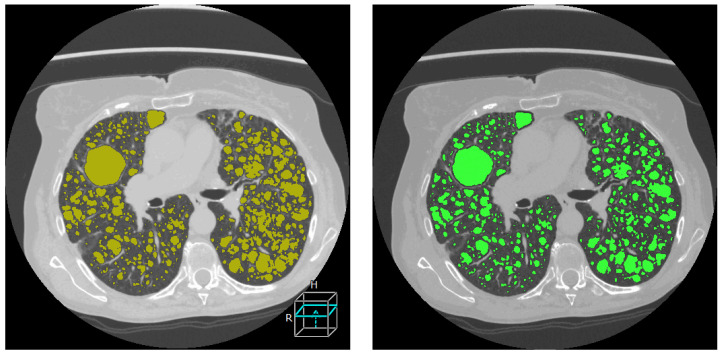
An example of the standard semi-automatic cyst segmentation (**left**) and fully automatic cyst segmentation (**right**) of an axial section of the chest CT scan of a patient with LAM. The cystic areas are highlighted in chartreuse color in the image on the left, and green color in the image on the right.

An example of pulmonary cyst segmentation by the standard semi-automatic method and by the automatic method is illustrated in Figure 2. The results of the statistical comparisons between the two methods are described in the sub-sections below.

### 3.1. Comparing Automatic and Standard Cyst Scores

**Figure 3:**
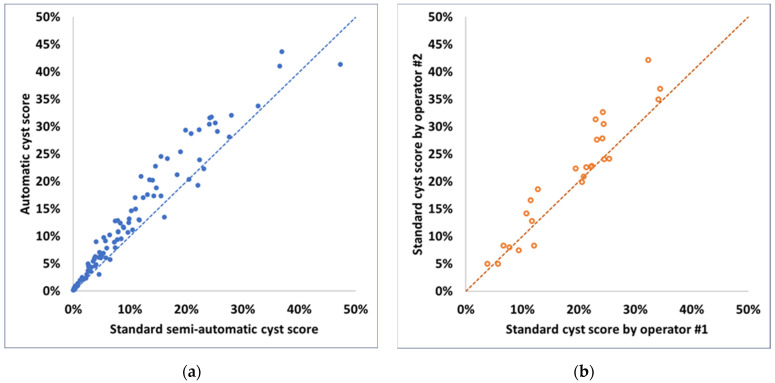
Scatter plots comparing the two pairs of cyst scores. (**a**) The automatic cyst scores versus the standard semi-automatic cyst scores of 100 chest CT scans from 100 patients. The dashed blue line is the identity line. (**b**) The standard semi-automatic cyst scores generated by trained operator #1 versus trained operator #2 for the same 26 chest CT scans from 19 patients. The dashed orange line is the identity line.

**Figure 4:**
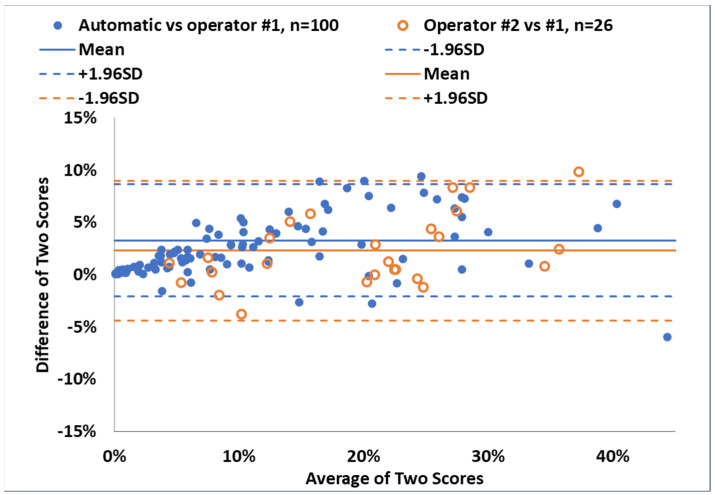
Bland–Altman plots of difference versus average value for the two pairs of cyst scores. The blue symbols and lines are the analysis of the difference between the standard semi-automatic cyst scores and the automatic cyst scores, both of 100 CT scans from 100 patients. The blue dots represent the difference versus the average for individual scans. The solid horizontal blue line represents the average of the differences, and the horizontal dashed blue lines represent the 95% confidence interval of the difference. Similarly, the orange symbols and lines are the analysis of the difference between a pair of standard semi-automatic cyst scores generated by operator #1 and operator #2, of 26 CT scans from 19 patients. The orange circles represent the difference versus the average for individual scans. The solid horizontal orange line represents the average of the differences, and the horizontal dashed orange lines represent the 95% confidence interval of the difference.

The standard semi-automatic cyst scores and the matching automatic cyst scores are represented in the scatter plot in Figure 3a. The standard scores on the same CT scans by two different trained operators are represented in Figure 3b. Both pairs of scores lay generally near the identity lines. The second pair covered a smaller range of cyst scores between 4.3% and 37.3%. In this range, the Bland–Altman analyses of the differences in the pairs of scores are summarized in Figure 4. The average difference between the automatic and the standard cyst scores was (mean ± std) (3.28 ± 2.72)%; the average difference between the two standard scores from the two operators was (2.29 ± 3.40)%. The two sets of differences did not differ from each other significantly either in their average values (*p* = 0.19) or in their variances (*p* = 0.078).

**Figure 5:**
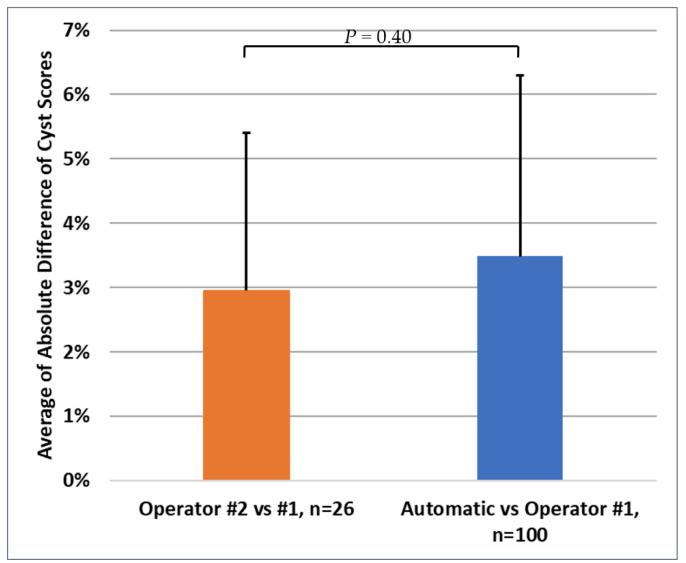
Average of the absolute value of the difference in the cyst scores generated by operator #1 vs. operator #2 (brown bar), and the average of the absolute value of the difference in the cyst scores generated by the automatic method vs. operator #1 (blue bar). The error bars represent the standard deviation. The two average values were statistically comparable (*p* = 0.40 for the comparison of the mean values and *p* = 0.19 for the comparison of the variances).

In terms of the absolute values of the difference between the automatic scores and the standard scores, the average of the absolute differences was (3.48 ± 2.46)%; it was (2.95 ± 2.81)% for the difference between the two standard scores from the two operators. These are summarized in Figure 5. Again, the two sets of absolute differences did not differ significantly from each other either in their average values (*p* = 0.40) or in their variances (*p* = 0.19).

### 3.2. Comparing the Correlation of the Cyst Scores to Pulmonary Function Tests

**Figure 6:**
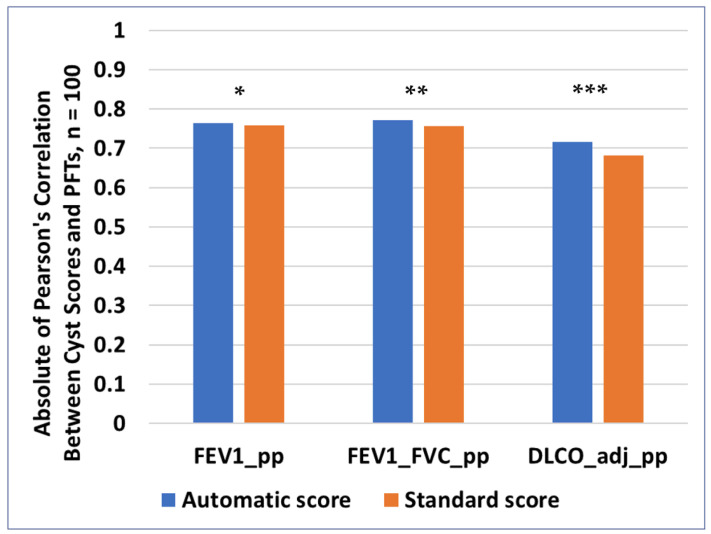
Absolute value of correlation between pulmonary function tests and the two types of cyst scores. The blue bars represent the cyst scores generated by the automatic method, and the orange bars represent the cyst scores generated by a trained operator using the standard semi-automatic method. The two sets of correlations were statistically comparable (*: *p* = 0.73; **: *p* = 0.36; ***: *p* = 0.062).

All the cyst scores were negatively correlated with the pulmonary function test results, meaning that higher cyst scores were associated with lower values of pulmonary function indices. The absolute values of Pearson’s correlation are summarized in Figure 6. The automatic cyst scores had a slightly stronger correlation with all three categories of PFTs compared to the standard semi-automatic scores, but the difference was not statistically significant. The correlation values were −0.765 vs. −0.760 for FEV_1__pp with *p* = 0.73 for the comparison, −0.772 vs. −0.757 for FEV_1_/FVC_pp with *p* = 0.36 for the comparison, and −0.715 vs. −0.681 for DL_CO__adj_pp with *p* = 0.062 for the comparison.

### 3.3. Comparing the Consistency of the Cyst Scores from the Two Methods

**Figure 7:**
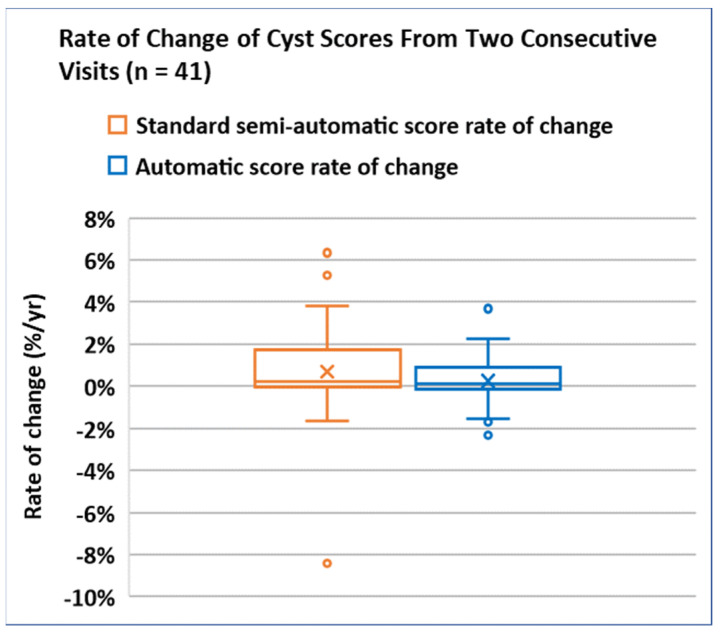
Box-and-whisker plot of the rate of change in the cyst scores obtained from two consecutive patient visits. The average rate of change for the standard semi-automatic (orange) and automatic (blue) methods were statistically comparable (*p* = 0.20). However, the spread of the rate of change in the automatic method was significantly smaller than that of the semi-automatic method (a standard deviation of 1.12%/year vs. 2.19%/year, *p* < 0.0001).

The consistency of the cyst scores was evaluated through the rate of change in the cyst scores over two consecutive visits. The results are summarized in Figure 7. The average rate of cyst score changes was (0.25 ± 1.12)%/year by the automatic method, and (0.68 ± 2.19)%/year by the standard semi-automatic method. The average value of the rate of change was not statistically different between the two methods with *p* = 0.20. The variance of the rate of change from the automatic method was significantly smaller than the one from the standard method (standard deviation of 1.12%/year vs. 2.19%/year, *p* < 0.0001).

## 4. Discussion

In cystic lung diseases such as lymphangioleiomyomatosis (LAM), a CT-based quantitative score of the extent of cystic changes in the lungs is often an integral part of the management of the disease which aids the evaluation of the stage of the condition and the effect of treatment [11,12,13]. Current FDA-approved software to segment the cysts in the CT images and generate the score is semi-automatic, where a trained operator visually adjusts the segmentation until it is deemed optimal. Although this procedure routinely provides clinically useful information, it is affected by inherent human subjective factors in the form of inter- and intra-operator inconsistencies [14], particularly when assessing changes over time. In response, we developed a fully automatic cyst segmentation and scoring method. In this study, the automatic method is validated against the standard semi-automatic method and against independent pulmonary function tests from the clinical physiology laboratory.

In the direct comparison between the automatic and the standard semi-automatic cyst scores, the difference between the standard cyst scores generated by the two operators independently was used as a reference. This reference provided a measure of the variability within the semi-automatic score itself. The results showed that the discrepancy between the automatic method and the standard method was as large as the discrepancy between the two trained operators running the same standard method. Therefore, the automatic method agreed with the standard method to the level allowed by the operator-related variability in the standard method. By the same reasoning, it is plausible that the difference between the automatic and semi-automatic scores could be accounted for by the human variability within the semi-automatic scores.

When it comes to the correlation between the cyst scores and pulmonary function as measured by the physiology laboratory tests, the automatic scores had slightly stronger associations with the PFT values compared to the standard semi-automatic scores, although the differences were not statistically significant (*p* ≥ 0.062).

In terms of the consistency of the cyst scores over time, we reasoned that inconsistency would lead to random fluctuations in the scores which would broaden the spread of the rate of change in the scores. Experimentally, the rate of change in the standard semi-automatic scores had about twice the spread of the one from the automatic scores (*p* < 0.0001). The evidence supports the notion that the automatic method improved the consistency of the cyst scores. This is expected, since the semi-automatic procedure is influenced by operator judgment, which may vary slightly between different operators and may fluctuate over time in the same operator. Therefore, by removing the human factor in the process, the level of consistency should improve when all the other factors are equal.

In the general context of the automatic segmentation of radiologic images in lung disease, machine learning-based approaches have been the focus of many efforts recently [22]. In the literature, we are aware of one attempt at developing a machine learning-based method to segment cysts in the chest CT scans of patients with LAM [23] which came from our institute. Although preliminary results showed promise, it is not yet fully functional. In contrast, the image-based adaptive method described in this study proved to be sufficiently robust to yield an operational software that is being used routinely in patients with LAM under a clinical research protocol at our hospital. The several experimental results in combination provide evidence that the automatic cyst segmentation and scoring serves as an equally accurate method as the FDA-approved standard semi-automated segmentation and scoring method. The results also suggest that the automatic method improved the consistency of the scores over time, which is an important factor when it comes to assessing the progression of the disease and the effect of treatment. A limitation of this study is the relatively small number of CT scans available to assess the variability within the standard method itself (26 scans from 19 patients). This point may be addressed in the future expansions of the study. Overall, it may be concluded that the automatic cyst score is a reliable replacement for the current gold-standard semi-automatic cyst score in the evaluation of patients with LAM disease.

## Data Availability

Please contact the corresponding author with reasonable requests for clinical study data.
